# Clinical outcomes of the EyeWatch system: three-year results

**DOI:** 10.1007/s00417-025-06942-2

**Published:** 2025-08-16

**Authors:** Constance Liegl, Sarah Hundertmark, Frank G. Holz, Karl Mercieca

**Affiliations:** Department of Ophthalmology, University of Bonn, Ernst-Abbe-Straße 2, Bonn, 53117 Germany

**Keywords:** Glaucoma surgery, EyeWatch system, Glaucoma drainage device

## Abstract

*****What is known***:**

- The EyeWatch™ System (EWS) is an adjustable glaucoma drainage device that allows non-invasive postoperative intraocular pressure (IOP) control.
- Early studies have shown promising short- and mid-term results with EWS in managing refractory glaucoma.

*****What is new***:**

- This study presents the first three-year clinical outcomes of EWS implantation, showing a sustained IOP reduction from a preoperative mean of 34.6 mmHg to 13.6 mmHg, with all patients achieving IOP levels below 21 mmHg and a significant decrease in medication burden.
- Most device adjustments occurred within the first postoperative year, after which IOP remained stable without further intervention.
- A transient IOP elevation following MRI underscores the importance of monitoring EWS patients in the context of magnetic exposure.

## Brief communications

**Fig. 1:**
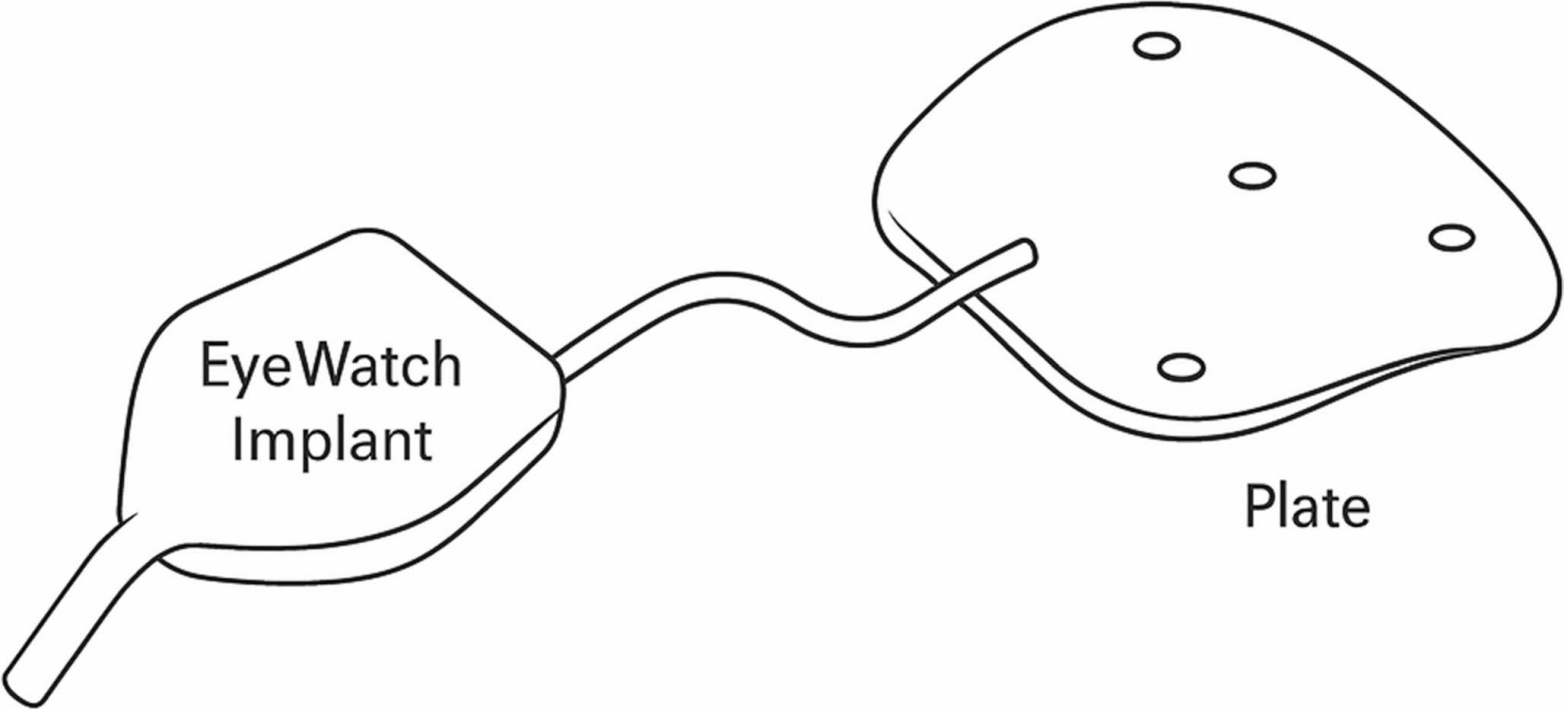
Schematic picture of the EWS with EyePlate

The eyeWatch™ System (EWS; Rheon Medical, Lausanne, Switzerland) offers an innovative solution for managing refractory glaucoma (Fig. 1). The EWS, used with a glaucoma drainage device (GDD), regulates aqueous flow via a magnetically adjustable deformable tube, allowing postoperative adjustment of intraocular pressure (IOP) without surgical intervention. This study presents three-year outcomes in five eyes undergoing EWS implantation (with 200 mm^2^ plate) at the University Hospital of Bonn.

All patients (mean age 66.2 years) presented with advanced glaucoma (2 primary open-angle glaucoma, 3 secondary glaucomas). Preoperative mean IOP was 34.6 mmHg (range: 23–45 mmHg) on 2.8 topical agents. At 12, 24, and 36 months, mean IOP declined to 12.2, 13.4, and 13.6 mmHg, respectively. The need for IOP-lowering agents decreased significantly, with all patients off acetazolamide postoperatively. Visual acuity remained stable or improved in most patients (Table 1).

**Table 1:** Demographic and clinical characteristics of patients who underwent EWS surgery

	Patient 1	Patient 2	Patient 3	Patient 4	Patient 5
Sex					
Male/Female	M	F	F	M	F
Ethnicity	Caucasian	Caucasian	Caucasian	Caucasian	Caucasian
Age	66	76	69	52	48
Glaucoma type	Secondary glaucoma due to panuveitis	POAG	POAG	Traumatic secondary glaucoma	Neovascular glaucoma due to pDRP
Previous vitrectomy	No	No	Yes	No	yes
Previous glaucoma surgery	No	Yes	No	Yes	no
Which one?		MP-CPC		XEN, trabectome	
Follow-up time	37	36	38	36	36
Anesthesia	GA	Local	GA	GA	GA
# of pressure-lowering eye drops	2	4	3	4	1
acetazolamide	no	no	no	yes	yes
Lens status	Pseudophakic	Pseudophakic	Pseudophakic	Pseudophakic	Phakic
BCVA (logMAR)	1.6	0.1	0.4	0.4	1.6
IOP preoperative	40	45	25	23	40
Maximum IOP without eye drops	41	48	31	41	40

EWS adjustments using the eyeWatch Pen™ (EWP) were performed in four of five cases, with 12 total adjustments in the first year. IOP decreased from a pre-adjustment mean of 21.3 mmHg to 8.6 mmHg after modification. No further adjustments were necessary after the first year.

Postoperative complications were limited. One patient required surgical revision due to tube exposure and inadequate IOP control, attributed to prior trauma and multiple surgeries. Minor complications included self-resolving hyphema (2 eyes) and a vitreous hemorrhage (1 eye). Notably, one case of transient IOP elevation followed an MRI scan; this was corrected by EWS adjustment. No vision-threatening events occurred.

Compared to Roy et al.‘s 2-year results (IOP 11.5 mmHg, 1.0 medications; 42 eyes), our cohort had higher baseline IOP but achieved comparable reductions (IOP 13.4 mmHg, 0.6 medications at 24 months) [1]. Roy et al. also compared EWS with Ahmed Glaucoma Valves (AGV) in a smaller study, showing a reduction from 27.3 mmHg to 12.8 mmHg in the EWS group at 12 months (12 AGV, 9 EWS combined with Baerveldt plate) [2]. Detorakis et al. described two patients with IOP reductions from 45 to 12 mmHg and 36 to 15 mmHg at one year; one patient did not respond despite valve opening [3]. The ability to modulate IOP non-invasively distinguishes EWS from traditional GDD such as Ahmed or Baerveldt implants, which rely on fixed resistance or surgical revisions [4]. Roy et al. reported 23 EWS adjustments in 8/9 patients during year one, aligning with our experience [5].

MRI compatibility is a unique consideration with the EWS [6]. In our cohort, one IOP spike occurred after cranial MRI. This phenomenon has been described in the literature and highlights the need for IOP monitoring post-MRI. EWS position was likely affected by magnetic interaction but was reversible.

This study confirms the EWS as an effective option for long-term IOP control with a low complication rate. Its non-invasive adjustability provides a significant advantage in early postoperative management. Limitations include the small sample size, lack of endothelial data, and retrospective design. Longer-term and comparative studies are warranted.

## Data Availability

All data is available from the authors.
